# Prevalence and factors associated with postpartum depression among Bhutanese mothers: a cross-sectional study

**DOI:** 10.4069/whn.2024.09.02

**Published:** 2024-09-30

**Authors:** Sherab Zangmo, Waraporn Boonchieng, Chalinee Suvanayos, Kelzang Gyeltshen, Pallop Siewchaisakul

**Affiliations:** 1 Faculty of Public Health, Chiang Mai University, Chiang Mai, Thailand; 2 Faculty of Nursing and Public Health, Thimphu, Bhutan; 3 Faculty of Nursing, Chiang Mai University, Chiang Mai, Thailand; 4 Jigme Dorji Wangchuck National Referral Hospital, Thimphu, Bhutan

**Keywords:** Bhutan, Depression, Postpartum period, Prevalence, Risk factors

## Abstract

**Purpose:**

This study investigated the prevalence of postpartum depression (PPD) and explored associated factors among mothers attending postnatal care in Bhutan.

**Methods:**

A cross-sectional study was conducted from August to November 2023 at a national referral hospital in Thimphu, the capital city of Bhutan. In total, 314 mothers were recruited. Sociodemographic, psychosocial, obstetric, and infant-related data were collected using questionnaires. The Edinburgh Postnatal Depression Scale, with a threshold of ≥11, was employed to screen for PPD, and logistic regression was used to test the potential factors.

**Results:**

The prevalence of PPD was 14.97%. Mothers with a perceived change in body image (adjusted odds ratio [AOR], 4.40; 95% confidence interval [CI], 1.91–10.17; *p*=.001), perceived heightened stress after delivery (AOR, 3.74; 95% CI, 1.45–9.67; *p*=.006), poor relationship with in-laws (AOR, 2.57; 95% CI, 1.24–5.30; *p*=.011), and negative birth experience (AOR, 2.42; 95% CI, 1.17–5.00; *p*=.016) demonstrated significantly higher odds of developing PPD. However, mothers with a higher monthly family income (Bhutanese ngultrum [Nu.] 20,000 to <50,000; AOR, 0.35; 95% CI, 0.13–0.92; *p*=.033), ≥Nu. 50,000 (AOR, 0.37; 95% CI, 0.13–1.07, *p*=.067) compared to <Nu. 20,000, and advanced gestational age (37 to <41 weeks; AOR, 0.25; 95% CI, 0.09–0.71; *p*=.009) and ≥41 weeks (AOR, 0.08; 95% CI, 0.00–0.75; *p*=.028) compared to <37 weeks had significantly lower risks of PPD.

**Conclusion:**

To mitigate the prevalence and risk of PPD, prioritizing screening strategies and interventions may benefit mothers with perceived changes in body image and heightened perceived stress after delivery, poor relationships with in-laws, and those with negative birth experiences.

## Introduction

Postpartum depression (PPD) is a nonpsychotic depressive disorder that affects mothers after childbirth. It is characterized by feelings of sadness, anxiety, diminished self-worth, changes in appetite, sleep, and mood, exhaustion, confusion, guilt, and difficulties in managing daily responsibilities. In severe cases, it can lead to suicidal thoughts [1,2]. These symptoms typically appear within the first 4 to 8 weeks after delivery, a period considered to be of high risk, although they can occur as late as 1 year postdelivery [3]. Mothers affected by PPD often struggle to provide optimal maternal care, which can lead to altered maternal behaviors and impaired maternal-infant bonding. These issues can have long-lasting impacts on the emotional, cognitive, and overall health of the infants, affecting interpersonal relationships, family dynamics, and social development [4,5]. If left undiagnosed and untreated, PPD can have complex effects on both mothers and infants. Moreover, persistent and severe cases of PPD may progress to postpartum psychosis, with statistics showing that 4% to 5% of affected women may commit infanticide or suicide [6]. Therefore, PPD represents a significant public health issue that poses substantial risks to maternal health and caregiving, with far-reaching consequences [7]. It is among the most common and debilitating mental health conditions affecting women of reproductive age [8].

The prevalence of PPD varies between countries and regions, with higher rates observed in developing countries. A pooled global prevalence of 17.22% has been reported [9]. Southeast Asian countries have higher prevalence rates than developed countries; with prevalence rates of 24.3% in Bangladesh [10], 22% in India [11], 19.3% in Pakistan [12], and 14.7% in Nepal [13]. Despite its prevalence, there remains a significant lack of studies, data, awareness, effective preventive measures, and intervention programs, particularly in developing countries [8,13]. Similarly, in Bhutan, mental illness is becoming a rising public health issue [14]. Between 2017 and 2021, the incidence of depression increased more than fivefold, from 6 per 10,000 to 32 per 10,000 population [15]. However, as of now, there are no published studies or official data on PPD and associated factors in Bhutan, to the best of our knowledge [16,17].

Although the main etiology of PPD remains still unknown [4], several factors have been consistently associated with its occurrence. These include sociodemographic factors such as the mother’s age, family income, education level, and family type [18]. In Bangladesh, a low maternal education level and low monthly income have been linked to an increased likelihood of experiencing PPD [10]. Psychosocial factors such as limited social support [19], history of violence or mental illness [20] maternal chronic illness [18], unplanned pregnancy [21], smoking, substance abuse [22], alcohol use [23], and perceived changes in body image after delivery [24], have also been identified as contributing to an increased risk of PPD. Additionally, stress perceived by mothers, along with their social isolation, were identified as risk factors [10]. Obstetric and infant-related factors, including parity [25], formula feeding [12], history of abortions, lower birthweight of the baby [26] cesarean births [27,28], a lack of prenatal checkups, unintended pregnancies, and preterm births were all associated with an increased risk of PPD [27-29]. However, factors such as having good social support, support from a spouse/partner [24], being married, breastfeeding [9], completing recommended prenatal care sessions, and having employed husbands/partners were inversely associated [18].

Bhutan has made remarkable progress in maternal healthcare, achieving a 98.4% rate of deliveries in healthcare facilities and a postnatal care coverage of 74% [15,30]. Postnatal mothers and their newborns receive follow-up care on days 3, 7, 21, and 42 to monitor their health [31]. Recently, Bhutan has introduced perinatal screening for depression, extending into the postpartum period. However, there is still a lack of statistical data and studies on PPD [16,17]. Therefore, the objective of this study was to ascertain the prevalence of PPD and identify the factors associated with it, in order to generate foundational, evidence-based data.

## Methods

**Ethics statement:** Ethical approval for this study was granted by the Research Ethics Committee of the Faculty of Public Health at Chiang Mai University, Thailand (ET020/2023), and by the Institutional Review Board of Khesar Gyalpo University of Medical Sciences in Bhutan (PN/2023-008/1052). All research protocols and procedures adhered to the relevant guidelines and regulations. Written consent was obtained from all participants, and for those under 18 years old, assent was also secured from their legal guardians.

### Study design

This cross-sectional study was conducted at the Gyaltsuen Jetsun Pema Wangchuck Mother and Child Hospital (GJPWMCH), a national referral hospital located in Thimphu, the capital city of Bhutan. The study adhered to the STROBE guidelines (https://www.strobe-statement.org/).

### Sample and sampling

Mothers who visited GJPWMCH for postnatal checkups constituted our study population. We invited all mothers who were between 4 to 8 weeks postdelivery and willing to provide written consent to participate. Non-Bhutanese mothers and those unable to respond for any reason were excluded. We employed a convenience sampling method to recruit participants.

**Figure 1.**
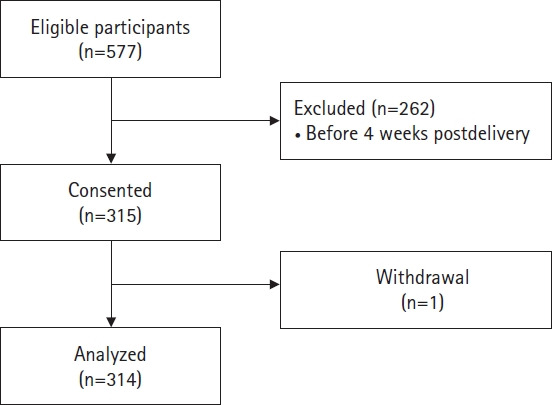
Flowchart of participant recruitment.

The sample size was calculated using a formula for a single population proportion, with the following assumptions: a reference prevalence of PPD in Bangladesh at 25% [10], a 95% confidence level, a 5% margin of error, and an additional 10% to account for potential incomplete data. Based on these calculations, the minimum required sample size was 285. To accommodate the risk of incomplete data, 315 eligible mothers were invited to participate, ensuring equal opportunity for all. After one participant withdrew, data from 314 postpartum mothers were analyzed (Figure 1).

### Measurements

Questionnaires were used to collect data; the 10-item Edinburgh Postnatal Depression Scale (EPDS) was employed for screening PPD, with the highest possible score ranging from 0 to 30, where a higher score indicates a depressive mood [1]. Researchers have utilized different EPDS thresholds, with averages ranging from 9 to 14 [18]. However, this study adopted an EPDS threshold of ≥11, as a recent comprehensive review and meta-analysis identified this threshold as providing the optimal balance between sensitivity (81%) and specificity (88%) [32]. The questionnaires were verbally translated into a simple and comprehensible local language and were pilot-tested on 30 Bhutanese postpartum mothers, who were not part of the main study. The standardized α-coefficient of the original EPDS was 0.87, indicating high reliability [1]. Cronbach’s alpha for the pilot test was .72, and for this study, it was .85.

Based on a comprehensive review of existing literature and with a focus on investigating potential factors associated with PPD, additional questionnaires were developed. These questionnaires included three domains: (1) sociodemographic factors such as age, education level, monthly family income, and type of family living arrangement; (2) psychosocial factors including social relationships, history of domestic violence, mental health issues, perceived stress during and after pregnancy, pregnancy intention, alcohol and drug use, and perceived changes in body image postdelivery; (3) obstetric and infant-related factors such as the number of living children, antenatal visits, mode of delivery, complications during antenatal, intrapartum, and postpartum periods, gestational age, and the birth weight of the baby. A panel of experts reviewed the questionnaires before implementation.

### Data collection and statistical analysis

The questionnaires were administered verbally by a trained research assistant in a separate room at GJPWMCH. The coded data were entered into an Excel spreadsheet and processed using STATA version 15.1 (StataCorp LLC). A descriptive analysis was conducted to compare the characteristics of participants with PPD using the chi-square test, and the results were presented in frequencies and percentages. Only significant variables were further analyzed using simple logistic regression. Independent variables with a significance level of *p*≤.05 were reexamined using multivariable logistic regression following a backward elimination process. Adjusted odds ratios (AORs) with 95% confidence intervals (CIs) and a *p*-value of ≤.05 as an indicator of statistical significance were utilized to assess the strength of associations.

## Results

### Characteristics of the participants

The participants’ ages ranged from 17 to 48 years, and 195 (62.10%) were between 25 and 35 years. More than half of the participants (n=176, 56.10%) had attained an education level of high school, and (n=174, 55.41%) did not have any direct income (Table 1). A significant number of participants (n=171, 54.46%) experienced a change in their body image after giving birth, and more than half of the participants reported heightened stress after delivery (n=190, 60.50%) (Table 2). Fewer than 50% of the participants had eight or more antenatal visits. Most deliveries occurred between 37 to <41 weeks of gestation (n=256, 81.52%), while negative birth experiences were expressed by (n=80, 25.48%) of participants (Table 3).

### Prevalence of postpartum depression

The prevalence of PPD was found to be 14.97% (95% CI, 11%–19%) when using a cutoff point of ≥11. The mean score was 14.17 (standard deviation, ±3.44).

Table 4 presents the response rates for each question on the EPDS, following participant regrouping. Responses were regrouped based on their original values, with scores of 0 and 1 categorized as “no” and scores of 2 and 3 categorized as “yes.” For Q10, a score of 0 was categorized as “no,” while scores of 1, 2, and 3 were categorized as “yes” [18]. The highest percentage of symptoms were reported for Q4 (78.72%) and Q3 (72.34%) relating to anxiety, whereas the lowest was noted for Q1 (1.50%) and Q2 (2.25%), relating to anhedonia. Q10 (7.01%), pertains to having thoughts of self-harm at least once within the last 1 week, which is concerning.

### Factors influencing postpartum depression

To examine the relationship between potential factors and PPD, twelve variables that were significant in the chi-square test were reanalyzed using simple logistic regression. These variables include monthly family income, relationship with friends, relationship with in-laws, history of mental health problems, perceived changes in body image, perceived stress during pregnancy, perceived stress after delivery, complications during pregnancy and delivery, overall birth experience, number of antenatal visits, gestational age, and the birthweight of the baby (Supplementary Table 1).

Table 5 presents the final model, which utilized multiple logistic regression with a backward elimination process. Participants who reported a perceived change in body image (AOR, 4.40; 95% CI, 1.91–10.17; *p*=.001), increased stress after delivery (AOR, 3.74; 95% CI, 1.45–9.67; *p*=.006), a poor relationship with their in-laws (AOR, 2.57; 95% CI, 1.24–5.30; *p*=.011), and a negative birth experience (AOR, 2.42; 95% CI, 1.17–5.00; *p*=.016) had higher odds of experiencing PPD. Conversely, participants with a higher monthly family income of ≥ Bhutanese ngultrum (Nu.) 20,000 to <Nu. 50,000 (AOR, 0.35; 95% CI, 0.13–0.92; *p*=.033) and >Nu. 50,000 (AOR, 0.37; 95% CI, 0.13–1.07; *p*=.067) compared to <Nu. 20,000, as well as those with advanced gestational ages of ≥37 to <41 weeks (AOR, 0.25; 95% CI, 0.09–0.71; *p*=.009) and ≥41 weeks (AOR, 0.08; 95% CI, 0.00–0.75; *p*=.028) compared to <37 weeks, significantly reduced the risk of PPD.

## Discussion

To our knowledge, this is the first study to identify the prevalence of PPD and associated factors among Bhutanese mothers. The research was conducted at the largest hospital in Thimphu, the capital city of Bhutan, and included participants from diverse backgrounds with varying educational levels and socioeconomic statuses. The prevalence of PPD was 14.97%, which is lower than that in Bangladesh at 24.3% [10] and China at 34% [19], but higher than that in Thailand at 10.4% [28]. These differences may stem from variations in research design, sample size, timing of screening, and outcome evaluation. For example, the study in Bangladesh was conducted at 24 weeks postpartum with an EPDS threshold of ≥10 [10]. In Sri Lanka, different prevalence rates have been determined for mothers at 10 days (15.5%) and 4 weeks postpartum (7.8%) using the same EPDS threshold of ≥9 [18]. Nonetheless, the prevalence of this study is comparable to that in Nepal (14.7%) [13]. Similarly, a comprehensive assessment of global PPD prevalence revealed a comparable rate of 13.53% for South-East Asia [9]. While the prevalence of 14.97% may not be notably high compared to other countries in the region, it remains significant and warrants concern for Bhutan.

In terms of factors associated with PPD, after adjusting for other variables, women who perceived a change in their body image postdelivery were more than four times as likely to experience PPD. This finding is consistent with a report indicating that PPD was more prevalent among obese women with limited social support compared to their counterparts in Korea [24]. In Ethiopia, overweight women also reported higher rates of PPD [33]. Similarly, in Spain, postpartum women who were dissatisfied with their body image tended to exhibit depressive symptoms more frequently [34]. However, a meta-analysis and comprehensive review identified only a weak association between body image dissatisfaction and PPD, despite consistent reports of the link [3]. To encourage a positive perception of body image changes after childbirth, it could be beneficial to inform and prepare mothers during the antenatal period and provide the necessary support to improve their body image postdelivery [34]. This study focused exclusively on the perceived feelings expressed by the participants, without considering their actual body weight or body mass index.

The finding that heightened perceived stress after delivery significantly increases the risk of PPD, with participants nearly four times more likely to be affected compared to those who reported no stress or a decrease in stress levels, aligns with previous studies from Bangladesh [10] and Iran [35]. Additionally, it has been noted that stressful life conditions, including significant life transitions, can contribute to PPD [3].

This study also observed that having a poor relationship with in-laws significantly increases the risk of PPD, with affected individuals facing more than twice the risk. This finding aligns with research conducted in India, which found that women ill-treated by their in-laws were nearly five times more likely to experience PPD [36]. A comprehensive study by Wang [9] revealed that nearly 80% of women with PPD had poor relationships with their mothers-in-law. Similar findings have been reported in other studies as well [37,38]. Strengthening the bond between a new mother and her mother-in-law has been shown to decrease the incidence of PPD in China [39]. A poor relationship with in-laws can be perceived as a diminished support system, exacerbating the emotional challenges and making women more susceptible to PPD. This is particularly significant as familial support is highly valued in Asian countries [3].

Our study found that participants who had negative birth experiences were more than twice as likely to experience PPD. Similarly, a study in Brazil reported that ill-treatment of women during childbirth was linked to a 50% increase in PPD symptoms [40]. Additionally, reduced satisfaction with the delivery process was identified as a strong indicator of PPD [41]. Consistent support throughout childbirth has been shown to decrease adverse experiences and improve outcomes for both mother and infant [42]. In Bhutan, approximately 12,000 to 13,000 women give birth annually, with 98.9% attended by skilled health professionals [15,43]. A previous study on respectful maternity care in Bhutan revealed that 41.8% of women had dreadful birth experiences [44]. Although the percentage of participants who reported negative birth experiences in this study was lower (25.48%), it remains a significant concern. The birth experience in this study may not fully reflect the care provided in health facilities but also includes support from husbands/partners and family members. This underscores the need for ongoing training of health professionals in comprehensive delivery care. Additionally, educating husbands/partners and family members about the importance of their support during this critical time is essential to ensure that women receive satisfactory care during birth and thereafter.

In addition to identifying risk factors, this study also highlighted factors that alleviate the risk. A higher monthly family income was inversely associated with PPD, mirroring findings from a Brazilian study where an improved financial status reduced the prevalence by nearly 50% [40]. Conversely, low family monthly income was linked with a raised probability of PPD in Indonesia [21] and Cameroon [20]. Furthermore, this observation is consistent with the World Health Organization’s STEPS survey in Bhutan, which emphasized socioeconomic factors as key elements in the mental health of women of reproductive age [45].

Advanced gestational age was identified as another mitigating factor, and similar findings were reported in Myanmar [29], and northwestern Ethiopia [26], where preterm delivery occurring before 37 weeks of gestation was inversely associated with PPD. A comprehensive review on preterm birth revealed that mothers of preterm babies are more likely to develop PPD compared to mothers of term babies [46]. This study found a preterm birth rate of 9.23%, slightly higher than that of a prior study (6.4%) [47]. The earlier study also noted that half of the preterm infants required admission to the neonatal intensive care unit. As the likelihood of newborn mortality and morbidity increases with decreasing gestational age, these factors might have contributed to the decreased risk of PPD as gestational age advances.

Although variables such as a history of mental health problems, inadequate antenatal visits, and complications during or after delivery did not reach statistical significance in the final model after adjusting for other factors, they were significant in the univariable analysis. Previous studies have reported associations between these variables and PPD, underscoring their potential importance: a history of mental health problems [23,35] not having the recommended number of antenatal checkups [18,29], and complications during/after delivery [38]. Therefore, further exploration of these factors and their relationship with PPD could provide a more comprehensive understanding.

A limitation of this study is that it examined various sociodemographic, psychosocial, and obstetric and infant-related factors, but did not include biological factors, which have been shown to be associated in previous studies. Additionally, the translation process of the EPDS was not independently verified; future studies should consider conducting psychometric testing. Furthermore, because this study was cross-sectional, it cannot establish causality or ensure generalizability.

In conclusion, nearly 15% of Bhutanese postpartum mothers experienced PPD. Mothers who perceived a change in body image, experienced increased stress after delivery, had poor relationships with their in-laws, or had negative birth experiences were at a greater risk of developing PPD. Conversely, a higher monthly family income and advancing gestational age were associated with a reduced risk of PPD. To mitigate the prevalence and reduce the risk of PPD, screening strategies and interventions should prioritize women with the aforementioned factors. Since these factors span all three domains, a multidisciplinary and comprehensive approach is essential. This study provides a foundational understanding of PPD and its associated factors among Bhutanese mothers, serving as a reference for future researchers. The integrated screening of perinatal depression with maternal and child health should be uniformly carried out, made accessible and available [5], and the involvement of healthcare professionals in perinatal screening should be enhanced to ensure that mothers at elevated risk for developing PPD are identified as early as possible [23]. Moreover, standard protocols on interventions are essential to guide health professionals in mitigating further risks and addressing associated implications.

## Figures and Tables

**Table 1.** Sociodemographic characteristics of participants with and without PPD (N=314)

Characteristics	Categories	n (%)			*p*-value
		Total	Normal^†^	PPD^‡^	
Age (year)^§^	<25	70 (22.29)	55 (78.57)	15 (21.43)	.126
	25–34	195 (62.10)	167 (85.64)	28 (14.36)	
	>35	49 (15.61)	45 (91.84)	4 (8.16)	
Education level	Primary school and below	56 (17.83)	53 (94.64)	3 (5.36)	.064
	High school	176 (56.10)	144 (81.82)	32 (18.18)	
	Others	82 (26.11)	70 (85.37)	12 (14.63)	
Religion	Buddhist	269 (85.70)	229 (85.13)	40 (14.87)	.905
	Others	45(14.33)	38 (84.44)	7 (15.56)	
Ethnicity	Ngalong	91(28.99)	77 (84.62)	14 (15.38)	.524
	Sharchop	136 (43.31)	112 (82.35)	24 (17.65)	
	Lhotsam	68 (21.66)	61 (89.71)	7 (10.29)	
	Others	19 (6.05)	17 (89.47)	2 (10.53)	
Currently living/address in Thimphu	No	58 (18.48)	47 (81.03)	11 (18.97)	.345
	Yes	256 (81.52)	220 (85.94)	36 (14.06)	
Employment status of husband/partner (N=313)	No	13 (4.14)	11 (84.62)	2 (15.38)	.966
	Yes	300 (95.54)	255 (85.00)	45 (15.00)	
Employment status of the participants	No	174 (55.41)	143 (82.18)	31(17.82)	
	Yes	140 (44.59)	124 (88.57)	16 (11.43)	.115
Type of employment	Government job	42 (13.38)	36 (85.71)	6 (14.29)	.387
	Private & co-operative	50 (15.92)	44 (88.00)	6 (12.00)	
	Housewife & unemployed	167 (53.18)	137 (82.04)	30 (17.96)	
	Others	55 (17.52)	50 (90.91)	5 (9.09)	
Monthly family income (Nu.)	<20,000	35 (11.15)	24 (68.57)	11 (31.43)	.015
	≥20,000, <50,000	182 (57.96)	159 (87.36)	23 (12.64)	
	≥50,000	97 (30.89)	84 (86.60)	13 (13.40)	
Type of family	Nuclear	150 (47.77)	133 (88.67)	17 (11.33)	.084
	Extended	164 (52.23)	134 (81.71)	30 (18.29)	

PPD: Postpartum depression. Bhutanese ngultrum (Nu.) 20,000=approximately 240 US dollars; Nu. 50,000=approximately 602 US dollars. Bold *p*-values mean statistical significance.

† Total Edinburgh Postnatal Depression Scale (EPDS) score of <11.

‡ Total EPDS score of ≥11.

§ Mean±SD=28.97±5.31 years (range, 17–48 years).

**Table 2.** Psychosocial characteristics according to the presence of PPD (N=314)

Characteristics	Categories	n (%)			*p*-value
		Total	Normal	PPD	
Relationship with husband	Good	212 (67.52)	184 (86.79)	28 (13.21)	.207
	Poor	102 (32.48)	83 (81.37)	19 (18.63)	
Relationship with family	Good	221 (70.39)	193 (87.33)	28 (12.67)	.078
	Poor	93 (29.61)	74 (79.57)	19 (20.43)	
Relationship with friends	Good	221 (70.38)	193 (87.33)	28 (12.67)	.043
	Poor	93 (29.62)	74 (79.57)	19 (20.43)	
Relationship with in-laws	Good	193 (61.46)	174 (90.16)	19 (9.84)	.001
	Poor	121 (38.54)	93 (76.86)	28 (23.14)	
Domestic violence, ever experienced	No	282 (80.81)	243 (86.17)	39 (13.83)	.093
	Yes	32 (10.19)	24 (75.00)	8 (25.00)	
Intimate partner violence, ever experienced	No	292 (93.00)	248 (84.93)	44 (15.12)	.856
	Yes	22 (7.00)	19 (86.36)	3 (13.64)	
Planned pregnancy	No	123 (39.17)	99 (80.49)	24 (19.51)	.070
	Yes	191 (60.83)	168 (87.96)	23 (12.04)	
Sex of the baby	Male	171 (54.46)	145 (84.80)	26 (15.20)	.898
	Female	143 (45.54)	122 (85.31)	21 (14.69)	
Preferred sex of the baby	No	51 (16.24)	43 (84.31)	8 (15.69)	.970
	Yes	111 (35.35)	94 (84.68)	17 (15.32)	
	No preferences	152 (48.41)	130 (85.53)	22 (14.47)	
Chronic diseases	No	285 (90.77)	243 (85.26)	42 (14.74)	.719
	Yes	29 (9.23)	24 (82.76)	5 (17.24)	
History of mental health problem	No	293 (93.31)	253 (86.35)	40 (13.65)	.015
	Yes	21 (6.69)	14 (66.67)	7 (33.33)	
Family history of mental health problem	No	299 (95.22)	255 (85.28)	44 (14.72)	.576
	Yes	15 (4.78)	12 (80.00)	3 (20.00)	
Perceived change in body image	No	143 (45.54)	133 (93.01)	10 (6.99)	<.001
	Yes	171 (54.46)	134 (78.36)	37 (21.64)	
Drinking alcohol	No	111 (35.35)	95 (85.59)	16 (14.41)	.839
	Yes	203 (64.65)	172 (84.73)	31 (15.27)	
Use of illegal drugs/substance abuse	No	310 (98.73)	264 (85.16)	46 (14.84)	.571
	Yes	4 (1.27)	3 (75.00)	1 (25.00)	
Smoking	No	257 (81.85)	222 (86.38)	35 (13.62)	.155
	Yes	57(18.15)	45 (78.95)	12 (21.05)	
Use of smokeless tobacco	No	297 (94.59)	254 (85.52)	43 (14.48)	.309
	Yes	17 (5.41)	13 (76.47)	4 (23.53)	
Chewing of betel quid	No	106 (33.76)	93 (87.74)	13 (12.26)	.354
	Yes	208 (66.24)	174 (83.65)	34 (16.35)	
Taking other forms of betel quid	No	157 (50.00)	139 (88.54)	18 (11.46)	.082
	Yes	157 (50.00)	128 (81.53)	29 (18.47)	
Perceived stress during pregnancy	No	97 (30.90)	89 (91.75)	8 (8.25)	.026
	Yes	217 (69.10)	178 (82.03)	39 (17.97)	
Perceived stress after delivery	No	124 (39.50)	118 (95.16)	6 (4.84)	<.001
	Yes	190 (60.50)	149 (78.42)	41 (21.58)	

PPD: Postpartum depression.

**Table 3.** Obstetric and infant-related characteristics according to the presence of PPD (N=314)

Characteristics	Categories	n (%)			*p*-value
		Total	Normal	PPD	
Number of living children	≤1	154 (49.04)	127 (82.47)	27 (17.53)	.211
	≥2	160 (50.96)	140 (87.50)	20 (12.50)	
Nature of labor pain	Spontaneous	149 (47.45)	120 (80.54)	29 (19.46)	.085
	Induced	78 (24.84)	68 (87.18)	10 (12.82)	
	Not gone into labor	87 (27.71)	79 (90.80)	8 (9.20)	
Any complications during or after delivery	No	286 (91.08)	247 (86.36)	39 (13.64)	.035
	Yes	28 (8.92)	20 (71.43)	8 (28.57)	
Admission of baby to a hospital/NICU (n=313)	No	190 (60.70)	162 (85.26)	28 (15.18)	.050
	Yes, for phototherapy	85 (27.16)	77 (90.59)	8 (9.41)	
	Yes, (others)	38 (12.14)	28 (73.68)	10 (26.32)	
Exclusive breastfeeding (n=313)	No	49 (15.65)	41 (85.42)	8 (16.67)	.282
	Yes	264 (84.35)	226 (85.61)	38 (14.39)	
Breastfeeding problem (n=313)	No	268 (85.62)	232 (86.57)	36 (13.43)	.142
	Yes	45 (14.38)	35 (77.78)	10 (22.22)	
Baby crying most of the time (n=313)	No	280 (89.56)	241 (86.07)	39 (13.93)	.059
	Yes	33 (10.54)	26 (78.79)	7 (21.21)	
Any congenital anomaly of the baby	No	295 (93.95)	253 (85.76)	42 (14.24)	.153
	Yes	19 (6.05)	14 (73.68)	5 (26.32)	
Overall delivery experience	Good	234 (74.52)	207 (88.46)	27 (11.540)	.004
	Bad/traumatic	80 (25.48)	60 (75.00)	20 (25.00)	
Number of ANC visits	<8	175 (55.73)	142 (81.14)	33 (18.86)	.030
	≥8	139 (44.27)	125 (89.93)	14 (10.07)	
Unfavorable obstetric history	No	284 (90.45)	239 (84.15)	45 (15.85)	.180
	Yes	30 (9.55)	28 (93.33)	2 (6.67)	
Mode of delivery of the baby	Normal delivery	169 (53.82)	140 (82.84)	29 (17.16)	.152
	Others^†^	145 (46.18)	127 (87.59)	18 (12.41)	
Gestational age (week)	<37	29 (9.24)	19 (65.52)	10 (34.48)	.003
	37-40	256 (81.52)	220 (85.94)	36 (14.06)	
	≥41	29 (9.24)	28 (96.55)	1 (3.45)	
Birth weight of the baby (g)	<2,500	39 (12.42)	28 (71.79)	11 (28.21)	.013
	≥2,500	275 (87.58)	239 (86.91)	36 (13.09)	

PPD: Postpartum depression; NICU: neonatal intensive care unit; ANC: antenatal care. Bold *p*-values mean statistical significance.

† Cesarean section and instrumental delivery.

**Table 4.** Response rate of participants for each question on the EPDS after regrouping (N=314)

EPDS questions	n (%)					
	Normal^†^		PPD^‡^		Total	
	No	Yes	No	Yes	No	Yes
1. I have been able to laugh and see the funny side of things	263 (98.50)	4 (1. 50)	39 (82.98)	8 (17.02)	302 (96.18)	12 (3.82)
2. I have looked forward with enjoyment to things	261 (97.75)	6 (2.25)	36 (76.60)	11 (23.40)	297 (94.59)	17 (5.41)
3. I have blamed myself unnecessarily when things went wrong	206 (77.15)	61 (22.85)	13 (27.66)	34 (72.34)	219 (69.75)	95 (30.25)
4. I have been anxious or worried for no good reason	200 (74.91)	67 (25.09)	10 (21.28)	37 (78.72)	210 (66.88)	104 (33.12)
5. I have felt scared or panicky for no very good reason	221 (82.77)	46 (17.23)	18 (38.30)	29 (61.70)	239 (76.11)	75 (23.89)
6. Things have been getting on top of me	231 (86.52)	36 (13.48)	17 (36.17)	30 (63.83)	248 (78.98)	66 (21.02)
7. I have been so unhappy that I have had difficulty sleeping	251 (94.01)	16 (5.99)	16 (34.04)	31 (65.96)	267 (85.03)	47 (14.97)
8. I have felt sad or miserable	255 (95.51)	12 (4.49)	19 (40.43)	28 (59.57)	274 (87.26)	40 (12.74)
9. I have been so unhappy that I have been crying	257 (96.25)	10 (3.75)	25 (53.19)	22 (46.81)	282 (89.81)	32 (10.19)
10. The thought of harming myself has occurred to me	264 (98.88)	3 (1.12)	28 (59.57)	19 (40.43)	292 (92.99)	22 (7.01)

EPDS: Edinburgh Postnatal Depression Scale; PPD: postpartum depression. For questions (Q) 1–9: a score of 0 & 1=No (normal symptoms) and score of 2 & 3=Yes (symptomatic). For Q10: a score of 0=No, and score of 1, 2, & 3=Yes.

† Total EPDS score of <11.

‡ Total EPDS score of ≥11.

**Table 5.** Factors associated with postpartum depression using multiple logistic regression following the backward elimination process: the final model (N=314)

Factors	Categories	AOR (95% CI)	*p*-value
Monthly family income (Nu.)	<20,000	1	
	≥20,000, <50,000	0.35 (0.13–0.92)	.033
	≥50,000	0.37 (0.13–1.07)	.067
Relationship with in-laws	Excellent/good	1	
	Strained/poor	2.57 (1.24–5.30)	.011
Perceived change in body image	No	1	
	Yes	4.40 (1.91–10.17)	.001
Perceived stress after delivery	No	1	
	Yes	3.74 (1.45–9.67)	.006
Overall delivery experience	Excellent/good	1	
	Bad/traumatic	2.42 (1.17–5.00)	.016
Gestational age (week)	<37	1	
	37-40	0.25 (0.09–0.71)	.009
	≥41	0.08 (0.00–0.75)	.028

AOR: Adjusted odd ratio; CI: confidence interval. Bhutanese ngultrum (Nu.) 20,000=approximately 240 US dollars; Nu. 50,000=approximately 602 US dollars.

## Supplementary materials

Further details on supplementary materials are presented online (available at https://doi.org/10.4069/whn.2024.09.02).

Supplementary Table 1. Association between potential factors and PPD using simple logistic regression (N=314)
