# Supplementary material for: Prevalence and factors associated with postpartum depression among Bhutanese mothers: a cross-sectional study
Source: Womens Health Nurs. 2024 Sep 30;30(3):238–49. doi: 10.4069/whn.2024.09.02 (PMC11467251; doi:10.4069/whn.2024.09.02)
Supplement: Supplementary Table 1. — Association between potential factors and PPD using simple logistic regression (N=314) [file whn-2024-09-02-Supplementary-Table-1.pdf]

Supplementary Table 1. Association between potential factors and PPD using simple logistic regression (N=314)

Factor	Categories	OR (95% CI)	*p*-value
Monthly family income (Nu.)	< 20,000	1	
	≥ 20,000, < 50,000	0.31 (0.13–0.72)	.007
	≥ 50,000	0.33 (0.13–0.84)	.021
Relationship with friends	Excellent/good	1	
	Strained/poor	1.91 (1.01–3.61)	.045
Relationship with in-laws	Excellent/good	1	
	Strained/poor	2.75 (1.46–5.20)	.002
History of mental health problems	No	1	
	Yes	3.16 (1.20–8.31)	.02
Perceived change in body image	No	1	
	Yes	3.67 (1.75–7.68)	.001
Perceived stress during pregnancy	No	1	
	Yes	2.43 (1.09–5.43)	.029
Perceived stress after delivery	No	1	
	Yes	5.41 (1.47–9.84)	< .001
Complications during and after delivery	No	1	
	Yes	2.53 (1.04–6.14)	.04
Overall delivery experience	Excellent/good	1	
	Bad/traumatic	2.55 (1.33–4.87)	.004
Number of antenatal visits	< 8	1	
	≥ 8	0.48 (0.24–0.94)	.033
Gestational age (week)	< 37	1	
	≥ 37, < 41	0.31 (0.13–0.72)	.007
	≥ 41	0.06 (0.01–0 .57)	.014
Birthweight of the baby (g)	< 2,500	1	
	≥ 2,500	0.38 (0.17–0.83)	.016

PPD: Postpartum depression; OR: odd ratio. CI: confidence interval.
Bhutanese ngultrum (Nu.) 20,000 = approximately 240 US dollars; Nu. 50,000 = approximately 602 US dollars.
$p \leq 0.05$, statistically significant.
